# Reduction in murine acute GVHD severity by human gingival tissue-derived mesenchymal stem cells via the CD39 pathways

**DOI:** 10.1038/s41419-018-1273-7

**Published:** 2019-01-08

**Authors:** Xuhao Ni, Yongxiang Xia, Shun Zhou, Hao Peng, Xiao Wu, Hao Lu, Han Wang, Rui Liu, Bruce R. Blazar, Jian Gu, Ling Lu

**Affiliations:** 1 Hepatobiliary Center, First Affiliated Hospital, Jiangsu Key Laboratory of Xenotransplantation, Collaborative Innovation Center for Cancer Medicine, Nanjing Medical University, 211166 Nanjing, Jiangsu China; 2 State Key Laboratory of Reproductive Medicine, Nanjing Medical University, 211166 Nanjing, Jiangsu China; 3 Department of General Surgery, Second Affiliated Hospital, Nanjing Medical University, 210000 Nanjing, Jiangsu China; 4 Division of Blood and Marrow Transplantation, Department of Pediatrics, University of Minnesota Masonic Cancer Center, University of Minnesota, Minneapolis, MN 55455 USA

## Abstract

Human gingival tissue-derived mesenchymal stem cells (GMSCs) present an accessible source of mesenchymal stem cells (MSCs) for treating autoimmune diseases. Here we show that human GMSCs can prevent and treat acute graft-versus-host disease (GVHD) in two different mouse models. Our results indicate that besides exhibiting suppressive function in vitro and in vivo, GMSCs may also regulate the conversion of Tregs to Th1 and/or Th17-like cells, as well as stabilize Foxp3 expression. Furthermore, GMSC-mediated prevention of acute GVHD was dependent on CD39 signaling that play an important role in the function and stability of Tregs. Finally, we also observed stronger protective ability of GMSCs with greater expansion ability compared with BMSCs or ASCs. These results indicate that human GMSCs have the potential to be used to treat GVHD.

## Introduction

Administration of mesenchymal stem cells (MSCs) represents a promising treatment for patients suffering from autoimmune disorders. Exogenous mesenchymal stem cells have been shown to inhibit T-cell proliferation^1^, as well as improve outcomes in preclinical murine models of GVHD^2^ and clinical steroid refractory GVHD in children^3^. Use of gingival-derived MSCs (GMSCs)—a population of stem cells that exists in the human gingival tissue—has several advantages over that of bone marrow stromal cells (BMSCs): easier isolation, better population homogeneity, and more rapid proliferation^4^.

Acute GVHD is a severe complication of allogeneic hematopoietic stem cells and solid organ transplantation that is associated with significant morbidity and mortality. Current strategies to treat acute GVHD do not produce long-lasting responses and vary greatly between different individuals^5^. Thus, developing effective GVHD prevention and treatment strategies is key to improve the state of transplantation medicine.

CD39 is an ectoenzyme that hydrolyzes ATP and adenosine diphosphate (ADP) into adenosine monophosphate (AMP). Located on the surface of endothelial cells and circulating platelets, CD39 plays a role in the suppressive function of human and mouse regulatory T cells (Tregs)^6^. Previous data from our laboratory demonstrated that CD39 signaling is involved in mediating the protective effect of GMSCs^7^. Here, we investigated the potential therapeutic effects of GMSCs and the role that CD39 plays in this GMSC-mediated GVHD attenuation. Our data show that human GMSCs have therapeutic potential in ameliorating lethal acute GVHD through adenosine receptors.

## Materials and methods

### Animals

BALB/c (H-2d), C57BL/6 (H-2b; termed B6), DBA/2 (H-2d), and B6D2F1 (H-2b/d) mice were purchased from Jackson Laboratory (Bar Harbor, ME). C57BL/6 Foxp3-GFP-knock-in mice were generously provided by Dr. Talil Chatilla (UCLA) and bred in our animal facility. Mice were used at age of 8–12 weeks. All murine experiments were performed in accordance with protocols approved by the Institutional Animal Care and Use Committees at University of Nanjing Medical University.

### GMSCs, BMSCs, and adipose stromal/stem cell (ASC) preparation

Human gingiva samples were collected following routine dental procedures at Nanjing Medical University, with approval by the Institutional Review Board. Human GMSCs were obtained as previously described^4^.

Human BMSCs were isolated by differential adhesion from a 30 mL BM aspirate obtained from the iliac crest of two human donors (Lonza, Hopkinton, MA) at the First Affiliated Hospital of Nanjing Medical University in China with approval by the ethics committee of Jiangsu People’s Hospital. Mononuclear cells (MNC) were enriched from the BM by using ACK Lysis Buffer (Lonza, Walkersville, MD) and long-term culture. The cells were cultured in MSC growth medium consisting of Minimum Essential Medium Alpha supplemented with 10% fetal bovine serum (Gibco, Grand Island, NY), 1% Penicillin-Streptomycin (Sigma Aldrich, St. Louis, MO), 2.5 μg/L FGF (R&D Systems, Minneapolis, MN), 2 ml/L Gentamicin (Sigma Aldrich, St. Louis, MO), and 2.2 g/L NaHCO3 (Sigma Aldrich, St. Louis, MO) at 37 °C with 5% carbon dioxide. On day 5, non-adherent cells were removed, and the growth media was fully replaced. Adherent cells were then expanded for another two weeks. Cells were washed with phosphate-buffered saline (PBS) (Thermo Fisher Scientific Waltham, MA), and the media was replaced on day 14.

### Adipose stromal/stem cell (ASC) preparation

Following ethics approval by Jiangsu People’s Hospital, human ASCs were isolated from donated subcutaneous lip aspirates and tissue from abdominoplasties of two donors using previously described methods^8,9^. Briefly, liposuction tissues were washed with PBS, digested for 1 h in PBS supplemented with 1% bovine serum albumin, 0.1% collagenase type 1 and 2 mM CaCl_2_. The stromal vascular fraction (SVF) was found in the pellet after centrifugation at 300 g at room temperature. The SVF cells were then expanded in DMEM/F12 Ham’s medium supplemented with 10% fetal bovine serum and 1% antibiotic/antifungal agents until >80% confluent. Adherent ASCs were dislodged from tissue culture flasks using trypsin digestion. The cells were characterized by cell surface immunophenotyping, as well as in vitro (data not shown).

### Induction of CD4^+^ Tregs in vitro

Naïve CD4^+^CD25^−^CD62L^+^ T cells were purified from the spleens of Foxp3-GFP C57BL/6 mice via magnetic isolation (Miltenyi Biotec). GMSCs or fibroblast cells were co-cultured with naïve CD4^+^CD25^−^CD62L^+^ T cells (1:5), and stimulated with beads coated with anti-CD3 and CD28 mAb (1:5) in the presence of IL-2 (100 IU/ml) and TGF-β (5 ng/ml) to induce Tregs. GMSCs and fibroblast cells were allowed to adhere to the plate overnight before the co-culture. In some experiments, rmIL-6 (10 ng/mL) and/or rmIL-1β (10 ng/mL) were also added. After 3 days, cells were harvested and analyzed by flow cytometry for **CD25**, **Foxp3**, and **CD39 expression**.

### Treg immunosuppression assays

WT naïve CD4+ T cells were labeled with CFSE and cultured in a 96-well bottom plate with anti-CD3/CD28-conjugated beads or anti-CD3-conjugated beads and APCs at a cell to bead ratio of 1:1. Serially diluted Treg cells were co-cultured for 72 h and cellular proliferation by CFSE was measured by flow cytometry.

### Acute GVHD models

Model I: BALB/c hosts were given total body irradiation (TBI; 800 cGy) from a 200-Kv x-ray source. Within 24 h, BALB/c mice were intravenously injected with 5 × 10^6^ T cell-depleted BM cells and 2 × 10^6^ T cells from C57BL/6 mice^10,11^. Mice were given medicated water (25 μg/ml neomycin/0.3 U/ml polymyxin B; Sigma-Aldrich)^12–16^. The survival of mice was monitored daily, and body weight was measured weekly.

Model II: Acute GVHD was induced by intravenous injection of 50 × 10^6^ splenocytes isolated from C57BL/6 mice into non-irradiated B6D2F1 mice as previously reported^17^. Mice were sacrificed after 2 weeks, and splenocytes were stained for the expression of H2k^b^, H2k^d^, and immune cell lineage markers using flow cytometry^18,19^.

### Chimerism assay

In Model II, mice were sacrificed on day 14, and the spleen cells were examined by flow cytometry. The proportion of donor to host cell was determined by staining H2-K^d^ and H2-K^b^ (donor cells are H2-K^b+/d−^; host cells are H2-K^b+/d+^).

### In vivo cytotoxic T cell activity

In vivo cytotoxic activity was determined using carboxyfluoroscein succinimidyl ester (CFSE, Thermo Fisher Scientific Waltham, MA) labeled target cells as described by manufacturer’s protocol. Briefly, spleen cells from DBA/2 mice were stained with 0.5 μM CFSE (CFSE^low^) and spleen cells from C57BL/6 mice were stained with 5 μM CFSE (CFSE^hi^). F1 mice were injected intravenously with a 1:1 mixture (1 × 10^7^ cells each) from both donors as target cells. CFSE staining density allowed distinction between injected DBA/2 and C57BL/6 cells. Five hours after adoptive cell transfer, mice were sacrificed. Their splenocytes were analyzed by flow cytometry to determine the percentage of CFSE^low^ and CFSE^hi^ cell populations. The absolute number of each target cell population was calculated based on the total spleen cell counts from individual mice. This number was multiplied by the percentage of CFSE^low^ and CFSE^hi^ cells as determined by flow cytometry for each respective mouse. The percent specific lysis was determined using frequency of donor cells: % lysis = (%CFSE^low^ in normal F1−% CFSE^low^ in experiments/%CFSE^low^ in normal F1)^20^.

### Flow cytometry

Spleen was prepared and stained with CD3 (Clone 17 A2), CD4 (Clone GK1.5), CD8 (Clone 53–6.7), CD19 (Clone 1D3), CD25 (Clone PC61), CD39 (Clone TU66), IFN-γ (Clone XMG1.2), TNF-α (Clone MP6-XT22), IL-17A (Clone N49-653), IL-4 (Clone 11B11), IL-10 (Clone JES5^−^16E3), IL-2 (Clone JES6-5H4), Helios (Clone 22F6), Fas (Clone Jo2), H2Kb (Clone AF6-88.5), H2Kd (Clone SF1-1.1.1) and analyzed on a FACSCalibour and a FACSLSR. Intracellular staining was performed with a Foxp3/Transcription Factor Staining Buffer Set (eBioscience).

### Statistical analyses

All data are represented as the mean ± SEM. Multiple regression and Student’s t-tests were used to determine statistical significance of non-survival data. Survival difference was determined by the Kaplan-Meier log-rank test. *P* < 0.05 was considered statistically significant.

## Results

### GMSC-induced iTregs exhibit suppressive function in vitro

**Fig. 1:**
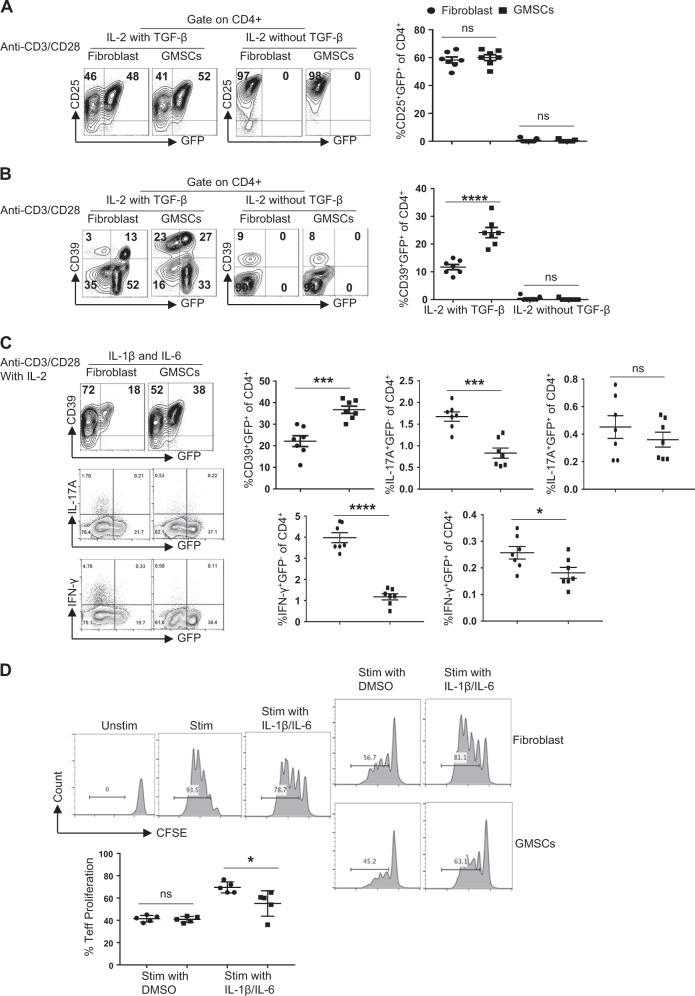
GMSCs stabilized Foxp3 expression and enhanced the suppressive function of CD4^+^ iTreg cells in the presence of IL-1β/IL-6 in vitro. **a** Naïve CD4 + T cells obtained from spleens were induced for 3 days with anti-CD3 mAb (2 ug/ml) coating the wells and free anti-CD28 mAb (2 ug/ml), IL-2 (100 U/ml), with or without TGF-β (5 ng/ml). Representative contour plots of CD25^+^Foxp3-GFP^+^ subset within CD4^+^ iTreg population in the presence of GMSC or fibroblast cells in vitro. Each symbol represents one independent experiments; ns, no significance, unpaired *t* test. **b** Representative contour plots of and CD39^+^Foxp3-GFP^+^ subset within CD4^+^ iTreg population in the presence of GMSC or fibroblast cells in vitro. Each symbol represents one independent experiments; ****P < 0.0001, ns no significance, unpaired *t* test. **c** iTreg cells induced in the presence of GMSCs or fibroblasts were expanded with IL-2, IL-1β and IL-6. Contour plots show CD39 vs. Foxp3-GFP, IL17A vs. Foxp3-GFP and IFN-γvs. Foxp3, the percentage of CD39^+^Foxp3-GFP^+^ cells, IL17A^+^Foxp3-GFP^+^ cells, IL17A^+^Foxp3-GFP^−^ cells, IFN-γ^+^Foxp3-GFP^+^ cells, and IFN-γ^+^Foxp3-GFP^−^ cells were measured. Each symbol represents one independent experiments; *P < 0.05, **P < 0.01, ***P < 0.001, ns, no significance, unpaired *t* test. **d** CFSE-labeled CD3^+^ T cells were co-cultured with both iTreg subsets at a 1:4 (2*104:8*104) ratio and anti-CD3 mAb-conjugated beads for 3 days. Cells were then stained with anti-mouse CD4 antibody and analyzed by flow cytometry. Each symbol represents one independent experiments; *P < 0.05, ns no significance, unpaired *t* test

Previous studies demonstrated that GMSCs can generate induced regulatory T cells (iTregs) in vivo without altering the natural regulatory T cell (nTreg) population^21^. iTregs have also been shown to inhibit murine acute GVHD^17^. Therefore, we tested the effect of GMSCs on iTreg generation in vitro. CD4^+^CD25^-^CD62L^+^ naïve T cells were purified from C57BL/6 Foxp3-GFP-knock-in mice and co-cultured with GMSCs or fibroblasts (1*10^6^:2*10^5^) (5:1) in the presence of anti-mouse CD3/CD28 mAb beads and IL-2 with or without TGF-β. GMSCs did not affect Foxp3 expression in CD4^+^CD25^+^ T cells cultured for 3 days independently of the presence of TGF-β in vitro (Fig. 1a). However, GMSCs increased the proportion of Foxp3-GFP^+^ cells co-expressing the CD39 ectonucleotidase when TGF-β was also present in the culture (Fig. 1b).

We next assessed the stability of iTregs in response to IL-1β and IL-6, two pro-inflammatory cytokines known to increase the degradation of Foxp3 and convert Tregs in effector T cells. After 3 days, iTregs were purified from the coculture containing GMSCs or fibroblasts using anti-CD4 mAb-conjugated beads. The cells were then cultured with DMSO or IL-1β and IL-6 for additional 3 days in the presence of IL-2. As predicted, Foxp3 expression was reduced in the fibroblast-containing iTreg group upon exposure to IL-1β and IL-6 (Fig. 1c), while the GMSC-containing iTreg group resisted Foxp3 downregulation. Also, the frequencies of IL-17A and IFN-γ in the fibroblast-containing iTreg group was higher than in the GMSC-containing iTreg group (Fig. 1c). The effect of IL-1β and IL-6 on iTreg function was also examined. iTregs purified as described above were co-cultured with CFSE-labeled CD4^+^ T cells (2*10^4^:8*10^4^) (1:4) for 3 days, and then analyzed by flow cytometry. Whereas iTregs previously cocultured with GMSCs and fibroblasts both showed similar ability to suppress T-cell expansion under normal conditions, only the fibroblast-induced iTregs lost their suppressive function following exposure to IL-1 and IL-6 (Fig. 1d).

### Administration of GMSCs improves survival and reduces pro-inflammatory cytokine production in acute GVHD in vivo

**Fig. 2:**
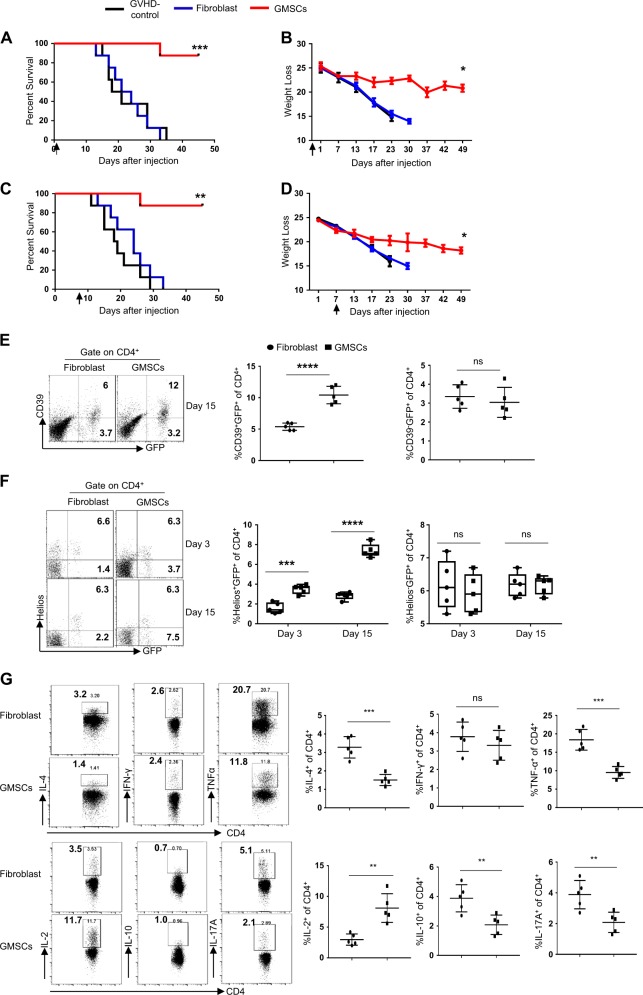
Injection of GMSCs, but not fibroblasts, decreased acute GVHD severity. Irradiated BALB/c mice were transplanted with T cell-depleted BM cells and CD3^+^ splenic T cells alone (BM only) with or without 2 × 10^6^ GMSCs or fibroblasts. **a** Recipient survival curve after GMSC or fibroblasts transfer on day 0. The number of mice is 8 for each group and three independent experiments was performed. ***P < 0.001, Log-rank (Mantel-Cox) Test. **b** Recipient weight loss curve after GMSC or fibroblasts transfer on day 0. the number of mice is 8 for each group and three independent experiments was performed. *P < 0.05, unpaired *t* test. **c** Recipient survival curve after GMSC or fibroblasts transfer on day 7. The number of mice is 8 for each group and three independent experiments was performed. **P < 0.01, Log-rank (Mantel-Cox) Test. **d** Recipient weight loss curve after GMSC or fibroblasts transfer on day 7. The number of mice is 8 for each group and three independent experiments was performed. *P < 0.05, unpaired *t* test. **e** CD39 vs. Foxp3-GFP expression in CD4^+^ T cells from the GMSCs group and Fibroblasts group. Each symbol represents one mouse and three independent experiments was performed. ****P < 0.0001, ns, no significance, unpaired *t* test. **f** Helios vs. Foxp3-GFP expression in CD4^+^ T cells from the GMSCs group and Fibroblasts group. Each symbol represents one mouse and three independent experiments was performed. ***P < 0.001; ****P < 0.0001, ns, no significance, unpaired *t* test. **g** Expression of IL-4, IL-17, IL-10, IFN-γ, IL-2, and TNF-α by CD4^+^ cells were determined by flow cytometry. Data are representative of three independent experiments. ***P < 0.001; ****P < 0.0001, ns no significance, unpaired *t* test

Next, we examined whether GMSCs can effectively eliminate acute GVHD in vivo. To accomplish this, two different acute GVHD models (C57BL/6-to-BALB/c and C57BL/6-to-B6D2F1) were used. In the C57BL/6-to-BALB/c model, mice were lethally irradiated and then rescued with an allogeneic donor BM. 2 × 10^6^ GMSCs or fibroblast cells were injected the same day as acute GVHD was induced. Mortality and weight loss were used to quantify progression of acute GVHD as previously described^22,23^. Recipients of GMSCs showed significantly improved survival (Fig. 2a, 87% vs. 0%, *P* < 0.0001) and weight loss (Fig. 2b; *P* < 0.031) compared to the non-treated or fibroblast-treated control groups. When GMSCs were injected 7 days after BM transplantation, a strong immunosuppressive environment ensued, with 87% long-term survival compared to 0% in the non-treated or fibroblast cell treated controls. These results indicated that exogenous GMSCs were effective in treating acute GVHD (Fig. 2b, 87% vs. 0%, *P* < 0.0025; Fig. 2d, *P* = 0.03). Because GMSCs enhanced CD39 expression in iTregs in vitro (Fig. 1) and Tregs can suppress acute GVHD^24^, we examined the frequency of CD39^+^ iTregs in this model. Compared with the fibroblast-treated group, the proportion of CD39^+^Foxp3^+^ cells was significantly increased in the GMSC-treated group on day 15 (Fig. 2e). Most of these Foxp3^+^ cells were Helios^−^iTregs, and no significant difference in the frequency of Helios^+^ nTregs was observed between the treatment groups on day 3 and day15 (Fig. 2f). We investigated why severity of acute GVHD was reduced in our murine models of the disease. Previous studies have suggested that cytokine production by donor cells contributed to the progression of acute GVHD^25,26^. To determine whether GMSCs could suppress acute GVHD through controlling cytokine production by staining with IFN-γ, IL-17, IL-4, TNF-α, IL-2, and IL-10, we detected the production of cytokines after injecting 15 days. Injection of GMSCs into mice with acute GVHD significantly reduced the percentages of cells secreting pro-inflammatory cytokines such as IFN-γ, IL-17, IL-4, and TNF-α. However, the IL-2 production was significantly increased in GMSCs group than Fibroblast group, which suggested that GMSCs can increased the differentiation and function of iTreg through enhancing the secretory of IL-2. Other Th2 cytokines such as IL-5 and IL-13 were undetectable in this model, and iTreg treatment did not alter their levels (data not shown) (Fig. 2g).

**Fig. 3:**
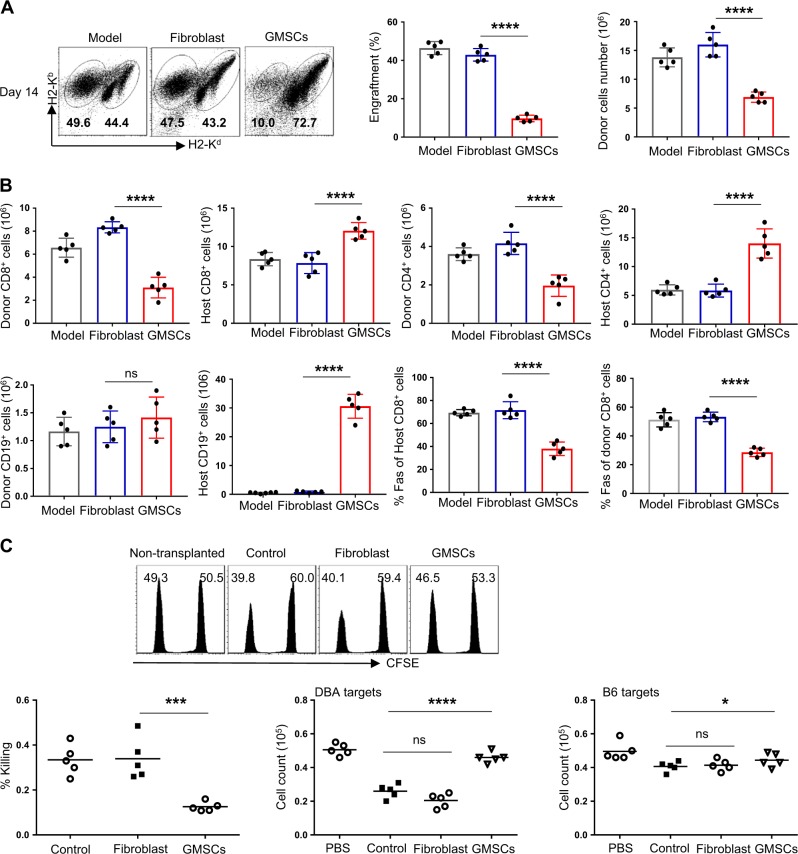
Adoptive transfer of GMSCs markedly suppressed the engraftment of donor cells in model two of acute-GVHD. Acute-GVHD (C57BL/6-to-F1) was induced as described in Methods. After two weeks, mice were sacrificed; donor and host splenic lymphocyte subsets were analyzed by flow cytometry. Donor and host cells can be distinguished by staining H2-Kd and H2-Kb—donor cells are H2-Kb^+^/d^−^ and host cells are H2-Kb^+^/d^+^. **a** H2-Kb vs. H2-Kd for donor and host cells after 14 days. Relative engraftment and absolute numbers of donor cells are shown for each group. Each symbol represents one mouse and two independent experiments was performed. ****P < 0.0001, unpaired *t* test. **b** Absolute number of donor CD8^+^ cells; host CD8^+^ cells, donor CD4^+^ cells; host CD4^+^ cells; donor CD19^+^ cells; host CD19^+^ cells; donor Fas^+^ in CD8^+^ cells; host Fas^+^ in CD8^+^ cells. Each symbol represents one mouse and two independent experiments was performed. ****P < 0.0001, ns, no significance, unpaired *t* test. **c** CSFE (CFSE^hi^ and CSFE^low^) was determined by Flow cytometry, and cells number from DBA targets and B6 targets was counted. Each symbol represents one independent experiments. *P < 0.0001, ***P < 0.0001, ****P < 0.0001, unpaired *t* test

In Model II of parent-into-F1 hybrid strain combination [C57BL/6-to-B6D2F1], 50 × 10^6^ C57BL/6 spleen cells were intravenously injected into non-irradiated immunocompetent D2B6F1 mice. After 14 days, mice were sacrificed, and the spleen cells were examined by flow cytometry. Donor and host cells can be distinguished by staining H2k^d^ and H2k^b^ since donor cells are H2k^b+^/k^d−^ and host cells are H2k^b+^/k^d+^. Nearly 50% of the administered donor cells were found in acute GVHD 2 weeks after adoptive cell transfer. Co-transfer of GMSCs markedly reduced donor engraftment and cell number (Fig. 3a). GMSCs suppressed the expansion of both donor CD4^+^ and CD8^+^ T cells. Host B cells, which are highly sensitive to depletion during active GVHD, were abundant in GMSC-treated mice compared to untreated GVHD controls and fibroblast-treated recipients (Fig. 3b). We attributed this observation to decreased FasL expression on donor CD8^+^ T cells and decreased Fas expression on host B cells (Fig. 3b) in GMSC-treated mice^27^.

To determine whether GMSCs suppressed the cytotoxic capacity of donor T cells, CFSE^hi^-labeled C57BL/6 and CFSE^low^-labeled DBA/2 splenocytes were mixed at a 1:1 ratio and administered in vivo with or without GMSCs^20,28^. After five hours, an approximately equal ratio of CFSE^hi^-labeled B6 and CFSE^low^-labeled DBA/2 splenocytes was recovered in the GMSC-treated group of non-transplanted F1 mice (Fig. 3c). In contrast, only 40% of DBA/2 cells were found in the untreated GVHD group. These results suggested selective killing of DBA/2 cells, while administration of GMSCs prevented the CTL priming response of F1 recipients against donor splenocytes.

### iTregs play a role in GMSC-mediated protection against acute GVHD in vivo

**Fig. 4:**
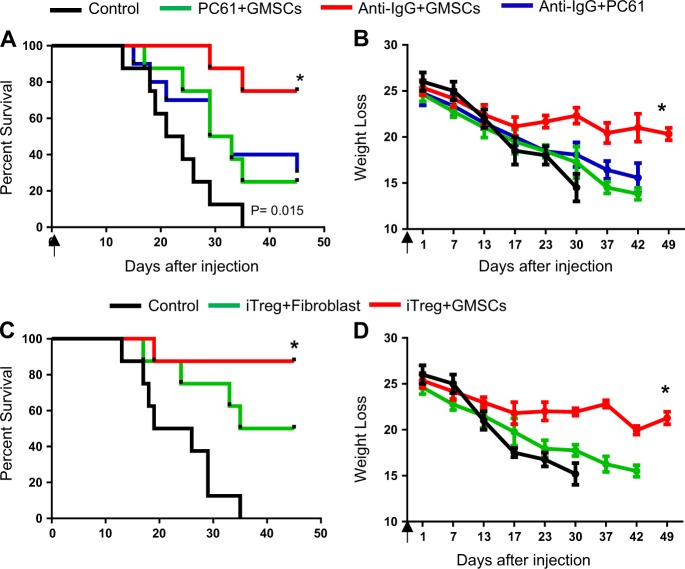
iTregs play a role in GMSC-mediated protection against acute GVHD in vivo. **a** Weight loss and (**b**) survival of acute GVHD mice that received GMSCs in the presence or absence of anti-CD25 mAb (clone PC61), which was administered to deplete Tregs. **c** Weight loss and (**d**) survival of acute GVHD mice receiving GMSC- or fibroblast-primed iTregs on day 0 are shown. The number of mice is 5 for each group and three independent experiments was performed. *P < 0.05; **P < 0.01; ****P < 0.0001, Log-rank (Mantel-Cox) Test for survival curve and unpaired *t* test for weight loss curve

To better understand the role of Tregs in GMSC-mediated protection against acute GVHD, we blocked Treg function by treating recipients in our GVHD Model I with an anti-CD25 antibody (clone PC61)^29,30^. PC61 was injected (IP) on the same day as BM and T cell transfer (IV) into the C57BL/6-to-BALB/c model. PC61 abrogated the protective effects of GMSCs during acute GVHD (Fig. 4a, 73% vs. 26%, *P* = 0.03; Fig. 4b, *P* = 0.025).

Because GMSCs enhanced CD4^+^ iTreg suppressive function without affecting Foxp3 expression in vitro (Fig. 1a), we studied the function of GMSC- and fibroblast-primed iTregs in vivo. GMSCs or fibroblasts were co-cultured with CD4^+^CD25^+^CD62L^+^ Naive T cells in the presence of TGF-β and IL-2 for 3 days to generate iTregs. Then, 2 × 10^6^ pretreated iTregs were purified by depleting the GMSCs or fibroblasts using anti-CD4-beads. These cells were injected into acute GVHD Model I. We observed that only GMSC-primed iTregs conferred protection against acute GVHD (Fig. 4c, d).

### CD39 pathways are involved in GMSC-induced GVHD attenuation

**Fig. 5:**
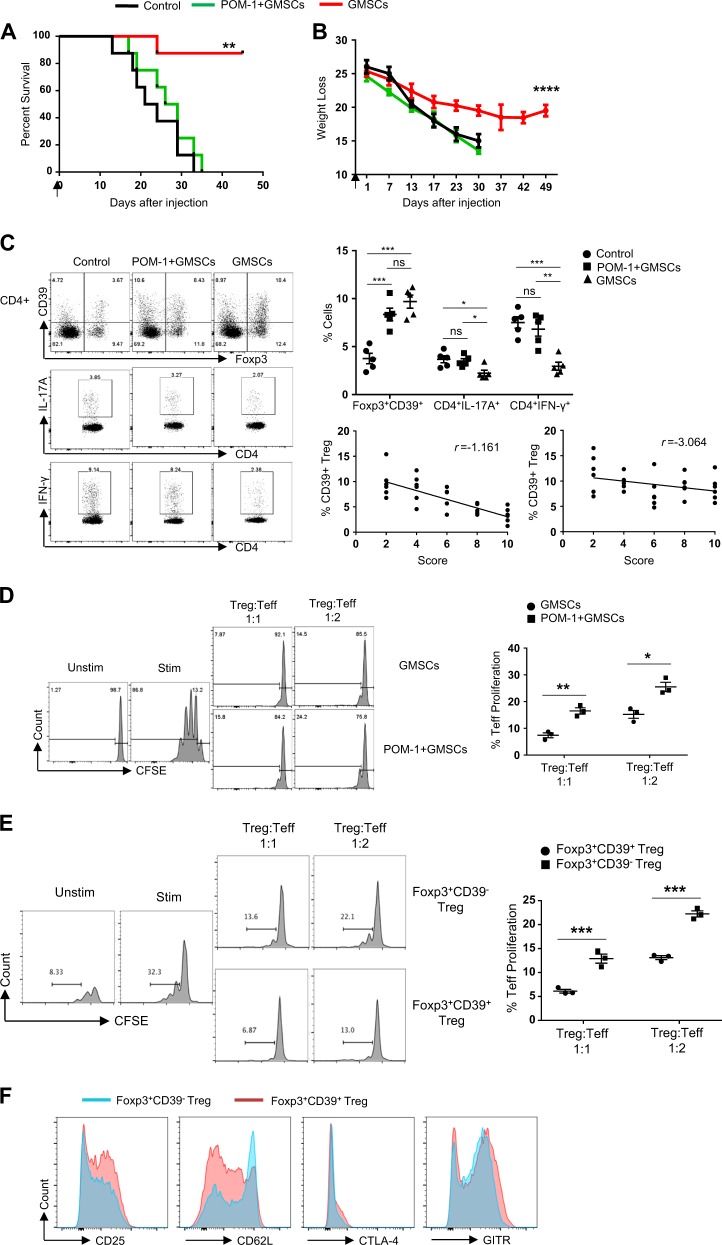
CD39 pathways are involved in GMSC-induced GVHD attenuation. **a** Weight loss and (**b**) survival of mice that received GMSCs pretreated or not treated with the CD39 inhibitor POM-1 for 4 h prior to injection. The number of mice is 5 for each group and three independent experiments was performed. Log-rank (Mantel-Cox) Test for survival curve and unpaired t test for weight loss curve. **P < 0.01; ****P < 0.0001. **c** The percentage of Foxp3^+^CD39^+^, IL-17A^+^ and IFN-γ^+^ in CD4 + T cells in spleen were measured by Flow Cytometry. The correlation between the frequency of CD39^+^/CD39^−^ Treg and the score of aGVHD was calculated by Linear regression-compare slopes. Each symbol represents one mouse and three independent experiments were performed unpaired t test. *P < 0.05; **P < 0.01; ***P < 0.001. **d** CFSE-labeled Naïve CD4 + T cells were co-cultured with GMSCs and POM-1 + GMSCs induced Tregs at a 1:1 and 2:1 ratio in the presence of anti-CD3/CD28 mAb-conjugated beads for 3 days. Cells were stained with anti-mouse-CD4 antibody and the suppressive activity of various primed subsets on CFSE-labeled CD4^+^ cells is shown. Each symbol represents one independent experiments, unpaired t test. *P < 0.05; **P < 0.01. **e** CFSE-labeled Naïve CD4 + T cells were co-cultured with Foxp3^+^CD39^+^ and Foxp3^+^CD39^+^ at a 1:1 and 2:1 ratio in the presence of anti-CD3 mAb-conjugated beads and APCs for 3 days. Cells were stained with anti-mouse-CD4 antibody and the suppressive activity of various primed subsets on CFSE-labeled CD4^+^ cells is shown. Each symbol represents one independent experiments, unpaired *t* test. **P < 0.01; ***P < 0.001. **f** The expression of CD25, CTLA-4, GITR, and CD62L were determined by Flow Cytometry and overlapped by Flowjo

Previous studies showed that POM-1, a CD39 inhibitor, diminished the ability of GMSCs to suppress T-cell expansion in vitro^21^. Consistently with this finding, our in vitro experiments demonstrate an increase in Foxp3^+^CD39^+^ iTregs in the presence of GMSCs (Fig. 1b). To confirm that CD39 is involved in GMSC-mediated GVHD attenuation, we assessed the effect of POM-1 treatment on GMSCs. The GMSCs were treated with POM-1 or isotype control for 4 h prior to injection into the GVHD Model I. We observed that the protective effect of GMSCs in acute GVHD was substantially reduced following treatment with POM-1 in vitro (Fig. 5a, b), suggesting that CD39 plays an important role in this process. Meantime, we measured the frequency of CD4^+^Foxp3^+^CD39^+^, CD4^+^IL-17^+^ and CD4^+^IFN-γ^+^ cells among control group, POM-1 + GMSCs group and GMSCs group. We found that GMSCs increased Treg function through the CD39 pathways (Fig. 5c). We further calculated the correlation between the frequency of CD39^+^ or CD39^−^ Treg and the score of aGVHD and found that only CD39^+^ Treg can affect the progression of aGVHD (Fig. 5c). In order to test whether POM-1 can abrogate the effect of GMSCs on Treg function. We used GMSCs and POM-1 + GMSCs induced Tregs to suppress the differentiation of Naïve CD4+ T cells in vito. We found that POM-1 + GMSCs induced Tregs failed to suppress the T cell responses (Fig. 5d). Meanwhile, we compared the immunosuppressive capacity between Foxp3^+^CD39^+^ Treg and Foxp3^+^CD39^−^ Treg by co-culturing CD4^+^ Naïve T cells labeled with CFSE and Foxp3^+^CD39^+^ Treg, Foxp3^+^CD39^−^ Treg sorted from Control group 3 days in present of anti-CD3 and APCs. The proliferation of CD4^+^ T cells was measured after staining with CD4, Foxp3^+^CD39^+^ Treg had more function than Foxp3^+^CD39^−^ Treg (Fig. 5e). We also examined the expression of those effector molecular that played a critical role in the Treg function between Foxp3^+^CD39^+^ Treg and Foxp3^+^CD39^−^ Treg, such as CD25, CTLA-4, GITR, CD62L. Our data shown that the expression of CD25, CTLA-4, and GITR in Foxp3^+^CD39^+^ Treg were higher than Foxp3^+^CD39^−^ Treg (Fig. 5f).

### GMSCs showed greater in vitro expansion capacity and stronger GVHD suppressive ability compared to BMSCs or ASCs

**Fig. 6:**
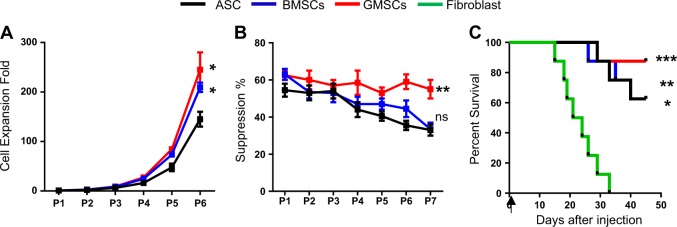
GMSCs showed greater in vitro expansion capacity and stronger GVHD suppressive ability compared to BMSCs and ASCs. **a** Absolute number of GMSCs, BMSCs, and ADCs after six passages in vitro. The result is representative of three independent experiments. *P < 0.05, unpaired *t* test. **b** CFSE-labeled Naïve CD4+ T cells were co-cultured with all three cell types for the indicated number of passages at a 20:1 ratio in the presence of anti-CD3 mAb-conjugated beads and APCs for 3 days. Cells were stained with anti-mouse-CD4 antibody and the suppressive activity of various primed subsets on CFSE-labeled CD4^+^ cells is shown. The result is representative of three independent experiments. **P < 0.01; ns, no significance, unpaired *t* test. **c** Survival following transfer of GMSCs, BMSCs, and ADCs on day 0. The number of mice is 8 for each group and three independent experiments was performed. *P < 0.05; **P < 0.01; ***P < 0.001, Log-rank (Mantel-Cox) Test

We and others have shown that human GMSCs, BMSCs, and ASCs display similar immunomodulatory properties such as inhibition of the activation and proliferation of human T cells. Because most MSCs are not functional at later passages and their expansion is limited, they lack potential for treatment of acute GVHD. Thus, we evaluated the expansion and suppression capabilities of GMSCs compared to BMSCs and ASCs by flow cytometry. GMSCs exhibited greater expansion following six in vitro passages compared to both BMSCs and ASCs (Fig. 6a). To evaluate the suppressive ability of these three cell types, each was co-cultured with mouse CD4^+^ Naïve T cells at a ratio of 1:20 at different passages. Irradiated non-T cells and anti-CD3 Ab were added. Proliferation was analyzed 3 days after T cell stimulation. Whereas all three mesenchymal stromal cell types inhibited CD4^+^ T-cell proliferation, GMSCs exhibited stronger suppressive ability compared to BMSCs and ASCs, especially after more passages (Fig. 6b). To test the function of the three cell subsets in vivo, we injected the three MSCs from passage 6 into the acute GVHD model. Although all three subsets elicited protection against acute GVHD, treatment with GMSCs resulted in highest survival rate (Fig. 6c).

## Discussion

GMSCs have been shown to be efficacious in different disease models, including collagen-induced arthritis^21^, colitis^31–33^, wound healing^34^, and Xeno-Graft-versus–Host Disease^35^. However, no group has yet reported that GMSCs can be used to treat acute GVHD. To answer this question, GMSCs were derived from human gingival tissues. Two distinct acute GVHD models were used to monitor GMSCs treatment. Our results clearly demonstrated that the superior effect of GMSCs in reducing the severity of acute GVHD generation and in treating mice with ongoing GVHD

Several factors may explain the mechanism by which GMSCs suppress acute GVHD. First, GMSCs reduced levels of several pro-inflammatory cytokines, particularly IL-17, IL-4, and IFN-γ, while increased levels of IL-2 which play a important role in the differentiation and function of iTreg. Second, GMSCs inhibited the proliferation of donor CD8 cells and reduced the cytotoxicity of donor CD8 cells to host CD19 cells. Furthermore, GMSCs reduced the expression of Fas on B cells and FasL on CD8 cells, which prevented killing of target B cells. Therefore, it appears that GMSCs protected the donor cells from apoptosis. Third, we detected higher expression of Foxp3 in GMSC-primed group compared to fibroblast controls in our acute GVHD model. Additionally, although GMSCs did not enhance Foxp3 expression in vitro, GMSCs increased the stability of Foxp3 expression and sustained the suppressive function of CD4 iTregs under inflammatory conditions.

Multiple studies have shown that the immunoregulatory function of GMSCs is associated with higher Treg frequency in vivo^36,37^. A population of Foxp3^+^CD39^+^ iTregs is highly expressed in TGF-β-generated CD4^+^ iTregs as a result of adding GMSCs to cell cultures in vitro; indeed, GMSCs were identified as having stronger regulatory function and stability^38^. We also detected higher Foxp3 expression in the GMSC-primed group compared to the Fibroblast-primed group, and most of the Foxp3^+^ cells were lacked Helios expression. In addition, our experiments showed that, although GMSCs had no effect on Foxp3 expression, GMSCs increased the stability of Foxp3 expression and sustained the suppressive function of CD4^+^ iTregs during the inflammatory response.

Recently, a population of CD4^+^CD39^+^Foxp3^+^ T cells was identified as having a regulatory function in the autoimmune disease model^39^. Our results suggest that Helios^−^ Foxp3^+^ T cells are a unique cell population that may be induced by GMSCs in acute GVHD. Interestingly, while the number of Treg cells as defined by Foxp3 expression did not increase, the survival of GMSC-treated group was much longer. As MSCs may not be able to infiltrate organs of interest, it is possible that soluble factors secreted by GMSCs may regulate Treg cell function.

In our previous study, we found that GMSCs treated collagen-induced arthritis through the CD39 pathway^22^. We examined whether CD39 is also an important pathway for GMSC treatment in acute GVHD. Transferred GMSCs pretreated with CD39 inhibitor (POM-1) had a reduced therapeutic effect in acute GVHD. Recent studies shown that expression of CD39 on CD4^+^CD25^+^Foxp3^+^ Tregs catalyzes the sequential generation of adenosine by degradation of extracellular ATP/ADP to CD39 and conversion of 5’-AMP to CD73^40,41^. These events lead to marked reduction in T-cell proliferation and secretion of pro-inflammatory cytokines. Herein, we demonstrated that GMSCs not only express CD39, but also increase the frequency of CD39^+^Foxp3^+^ Tregs, which supports the generation of adenosine and promotes immune suppression of effector T cells in vitro and vivo.

Together, our data indicate that GMSCs have a greater potential therapeutic effect against acute GVHD than BMSCs or ASCs. Adoptive transfer of GMSCs provided the clinical outcome in our acute GVHD model using older passaged cells compared to these other mesenchyme stromal cell types. Efficiency of using older passaged cells means that more cells can be made available for treatment.

These experiments suggest that human GMSCs selectively promote the Foxp3^+^CD39^+^ subset of iTregs. The ability of GMSCs to increase survival and reduce severity of acute GVHD appears to be associated with the up-regulation expansion, differentiation of Tregs in vivo through the CD39 pathways. Human GMSCs present a novel therapeutic strategy for preventing and treating acute GVHD, as well as autoimmune diseases. Given the promising results outlined above, future studies in the clinic will be needed to assess the true prophylactic and therapeutic potential of this readily accessible and unique source of MSCs.
